# *In silico* Analyses of Immune System Protein Interactome Network, Single-Cell RNA Sequencing of Human Tissues, and Artificial Neural Networks Reveal Potential Therapeutic Targets for Drug Repurposing Against COVID-19

**DOI:** 10.3389/fphar.2021.598925

**Published:** 2021-02-26

**Authors:** Andrés López-Cortés, Patricia Guevara-Ramírez, Nikolaos C. Kyriakidis, Carlos Barba-Ostria, Ángela León Cáceres, Santiago Guerrero, Esteban Ortiz-Prado, Cristian R. Munteanu, Eduardo Tejera, Doménica Cevallos-Robalino, Ana María Gómez-Jaramillo, Katherine Simbaña-Rivera, Adriana Granizo-Martínez, Gabriela Pérez-M, Silvana Moreno, Jennyfer M. García-Cárdenas, Ana Karina Zambrano, Yunierkis Pérez-Castillo, Alejandro Cabrera-Andrade, Lourdes Puig San Andrés, Carolina Proaño-Castro, Jhommara Bautista, Andreina Quevedo, Nelson Varela, Luis Abel Quiñones, César Paz-y-Miño

**Affiliations:** ^1^ Centro de Investigación Genética y Genómica, Facultad de Ciencias de la Salud Eugenio Espejo, Universidad UTE, Quito, Ecuador; ^2^ RNASA-IMEDIR, Computer Science Faculty, University of A Coruna, A Coruña, Spain; ^3^ Latin American Network for the Implementation and Validation of Clinical Pharmacogenomics Guidelines (RELIVAF-CYTED), Madrid, Spain; ^4^ One Health Research Group, Faculty of Medicine, Universidad de Las Américas (UDLA), Quito, Ecuador; ^5^ Heidelberg Institute of Global Health, Faculty of Medicine, Heidelberg University, Heidelberg, Germany; ^6^ Instituto de Salud Pública, Facultad de Medicina, Pontificia Universidad Católica del Ecuador, Quito, Ecuador; ^7^ Tropical Herping, Quito, Ecuador; ^8^ Biomedical Research Institute of A Coruna (INIBIC), University Hospital Complex of A Coruna (CHUAC), A Coruña, Spain; ^9^ Centro de Información en Tecnologías de la Información y las Comunicaciones (CITIC), A Coruña, Spain; ^10^ Grupo de Bio-Quimioinformática, Universidad de Las Américas (UDLA), Quito, Ecuador; ^11^ Hospital General del Sur de Quito, Instituto Ecuatoriano de Seguridad Social, Quito, Ecuador; ^12^ Faculty of Medicine, Pontifical Catholic University of Ecuador, Quito, Ecuador; ^13^ Carrera de Medicina, Facultad de Ciencias de la Salud Eugenio Espejo, Universidad UTE, Quito, Ecuador; ^14^ Centro Clínico Quirúrgico Ambulatorio Hospital del Día El Batán, Instituto Ecuatoriano de Seguridad Social, Quito, Ecuador; ^15^ Department of Plant Biology, Faculty of Natural Resources and Agricultural Sciences, Swedish University of Agricultural Sciences, Uppsala, Sweden; ^16^ Fundación Futuro, Quito, Ecuador; ^17^ Facultad de Ingeniería y Ciencias Aplicadas-Biotecnología, Universidad de Las Américas, Quito, Ecuador; ^18^ Laboratory of Chemical Carcinogenesis and Pharmacogenetics, Department of Basic-Clinical Oncology, Faculty of Medicine, University of Chile, Santiago, Chile

**Keywords:** COVID-19, immune system, single-cell RNA sequencing, artificial neural networks, drug repurposing

## Abstract

**Background:** There is pressing urgency to identify therapeutic targets and drugs that allow treating COVID-19 patients effectively.

**Methods:** We performed *in silico* analyses of immune system protein interactome network, single-cell RNA sequencing of human tissues, and artificial neural networks to reveal potential therapeutic targets for drug repurposing against COVID-19.

**Results:** We screened 1,584 high-confidence immune system proteins in ACE2 and TMPRSS2 co-expressing cells, finding 25 potential therapeutic targets significantly overexpressed in nasal goblet secretory cells, lung type II pneumocytes, and ileal absorptive enterocytes of patients with several immunopathologies. Then, we performed fully connected deep neural networks to find the best multitask classification model to predict the activity of 10,672 drugs, obtaining several approved drugs, compounds under investigation, and experimental compounds with the highest area under the receiver operating characteristics.

**Conclusion:** After being effectively analyzed in clinical trials, these drugs can be considered for treatment of severe COVID-19 patients. Scripts can be downloaded at https://github.com/muntisa/immuno-drug-repurposing-COVID-19.

## Introduction

The first zoonotic transmission of the severe acute respiratory syndrome coronavirus 2 (SARS-CoV-2) was located in China in December 2019 (Tay et al., 2020), and it is the causative agent of the coronavirus disease 2019 (COVID-19) (Sanders et al., 2020). The World Health Organization (WHO) declared the outbreak of COVID-19 as a Public Health Emergency of International Concern on January 30, 2020, and a pandemic on March 11, 2020 (Gao Q et al., 2020). Classified in the *Coronaviridae* family and *Betacoronavirus* genus, SARS-CoV-2 is the seventh CoV known to infect humans, along with 229E, NL63, OC43, HKU1, SARS-CoV, and Middle East respiratory syndrome (MERS) (Oberfeld et al., 2020). Coronaviruses cause mild to severe respiratory diseases and have high mutation rates that result in high genetic diversity, plasticity, and adaptability to invade a wide range of hosts (Peiris et al., 2004).

**FIGURE 1:**
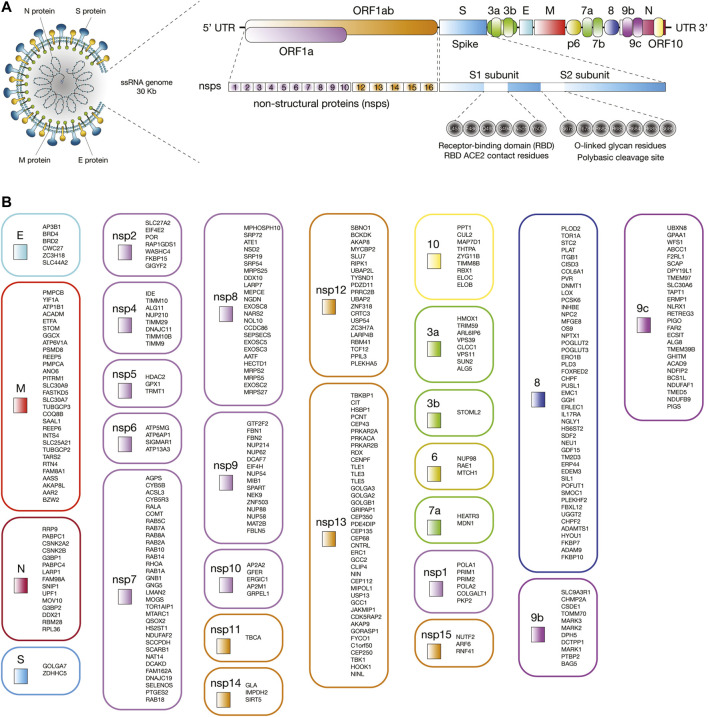
Interaction between human proteins and SARS-CoV-2 proteins. **(A)** Proteomic and genomic structure of SARS-CoV-2. **(B)** Human proteins physically associated with SARS-CoV-2 proteins.

The first genome of SARS-CoV-2 named Wuhan-Hu-1 (NCBI reference sequence NC_045512) was isolated and sequenced in China in January 2020 (Zhou P et al., 2020; Zhu et al., 2020). SARS-CoV-2 is a single-stranded positive-sense RNA virus of about 30 kb in length (Zhou P et al., 2020; Ziegler et al., 2020). The genomic structure is comprised of a 5′ terminal cap structure, 14 open reading frames (ORFs) encoding 29 proteins, and a 3′ poly A tail (Wu A et al., 2020). ORF1a and ORF1ab are the largest genes and codify 16 non-structural proteins (nsp1 to nsp16). According to Gordon et al. (2020), nsps are involved in antiviral response (nsp1), viral replication (the nsp3-nsp4-nsp6 complex), the protease 3C^pro^ (nsp5) (Zhang L et al., 2020), the RNA polymerase (the nsp7-nsp8 complex), the single-strand RNA binding (nsp9), the methyltransferase activity (nsp10 and nsp16), the RNA-dependent RNA polymerase (nsp12) (Gao Y et al., 2020), the helicase/triphosphatase (nsp13), the 3′-5′ exonuclease (nsp14), the uridine-specific endoribonuclease (nsp15), and the RNA-cap methyltranspherase (nsp16) (Gordon et al., 2020). Lastly, the 3′ terminus contains genes that codify the spike (S) glycoprotein, the envelope (E) protein, the membrane (M) glycoprotein, the nucleocapsid (N) protein, and several accessory proteins (3a, 3b, p6, 7a, 7b, 8, 9b, 9c, and 10) (Figure 1A) (Wu A et al., 2020; Wu C et al., 2020).

COVID-19 is caused when SARS-CoV-2 exploits the host cell machinery for its own replication and spread (Ortiz-Prado et al., 2020). SARS-CoV-2 entry into human cells is mediated by the S glycoprotein that forms homotrimers protruding from the viral surface (Walls et al., 2020). S1 and S2 are two functional subunits of the S glycoprotein. Six receptor-binding domain (RBD) amino acids (L455, F486, Q493, S494, N501, and Y505) of the S1 subunit directly bind to the peptide domain of angiotensin-covering enzyme 2 (ACE2) human receptor protein (Andersen et al., 2020; Cao et al., 2020; Wang Q et al., 2020; Yan et al., 2020). The affinity constant for RBD of SARS-CoV-2 to ACE2 is greater than that of SARS-CoV by as much as a factor of 10–15 (Wang Q et al., 2020, Wang Y et al., 2020; Wrapp et al., 2020). S glycoprotein is cleaved by the cathepsin L (CTSL) protease (Muus et al., 2020), and the transmembrane serine protease (TMPRSS2) in a functional polybasic (furin) cleavage site at the S1-S2 boundary flanked for O-linked glycans (Hoffmann et al., 2020; Walls et al., 2020). S2 subunit mediates subsequent fusion between the human and viral membranes (Kirchdoerfer et al., 2016; Yuan et al., 2017).

ACE2 is a type I membrane protein widely expressed in nasal goblet secretory cells, lung type II pneumocytes, ileal absorptive enterocytes, kidney proximal tubule cells, gallbladder basal cells, among other human cells (Deng et al., 2020; Lamers et al., 2020; Singh et al., 2020; Sungnak et al., 2020; Ziegler et al., 2020), and participates in the maturation of angiotensin, a peptide hormone that controls blood pressure and vasoconstriction (Donoghue et al., 2000). After virus entry, many severe ill COVID-19 patients developed clinical manifestations such as cough, mild fever, dyspnea, lung edema, severe hypoxemia, acute respiratory distress syndrome (ARDS) (Montenegro et al., 2020), acute lung injury (Blanco-Melo et al., 2020), interstitial pneumonia, increased concentrations of fibrinogen and D-dimer plasma levels (Spiezia et al., 2020; Tang et al., 2020), elevated levels of pro-inflammatory chemokines and cytokines such as interleukin (IL) 6 (Herold et al., 2020; Sarzi-Puttini et al., 2020), low levels of type I and III interferons (IFNs) (Blanco-Melo et al., 2020), high levels of lactate dehydrogenase, hyperferritinemia, idiopathic thrombocytopenic purpura caused by spleen atrophy (Zulfiqar et al., 2020), formation of hyaline membrane (Yao et al., 2020), hilar lymph node necrosis, lymphopenia (Terpos et al., 2020), intravascular coagulopathy (Fogarty et al., 2020), pulmonary thromboembolism (Rotzinger et al., 2020), hypotension (Rentsch et al., 2020), cerebrovascular events (Mao et al., 2020), severe metabolic acidosis, kidney and hepatic dysfunctions (Zhang C et al., 2020), secondary infections, septic shock (Li H et al., 2020), and multi-organ failure (Wang Q et al., 2020; Gupta et al., 2020; Wadman et al., 2020).

Additionally, SARS-CoV-2 interacts with the immune system triggering dysfunctional immune responses to COVID-19 progression (Tay et al., 2020). Given that an excessive inflammatory response to the novel coronavirus is thought to be a major cause of disease severity and death (Blanco-Melo et al., 2020; Mehta et al., 2020), a better understanding of the immunological underpinnings is required to identify potential therapeutic targets. To fill in this gap, we performed *in silico* analyses of immune system protein-protein interactome (PPi) network, single-cell RNA sequencing (scRNA-seq) of human tissues, and artificial neural networks to reveal potential therapeutic targets for drug repurposing against COVID-19.

## Methods

### Protein Sets

We have retrieved the 332 human proteins physically associated with 26 of the 29 SARS-CoV-2 proteins proposed by Gordon *et al* (Figure 1B; Supplementary Table S1; Gordon et al., 2020). We have also retrieved a total of 3,885 immune system proteins from several databases such as the International ImMunoGeneTics information system (http://www.imgt.org) (Giudicelli et al., 2005; Lefranc et al., 2009; Lefranc et al., 2015), the InnateDB database (https://www.innatedb.com/) (Breuer et al., 2013), and the David Bioinformatics Resource (https://david.ncifcrf.gov/) (Huang et al., 2009b; Huang et al., 2009a) using the gene ontology (GO) terms: 0002376 immune system process, 0045087 innate immune response, and 0002250 adaptive immune response. Lastly, both protein sets were integrated to identify the highest confidence interactions and to design the immune system PPi network.

### Protein-Protein Interactome Network

The immune system PPi network with a highest confidence cutoff of 0.9 and zero node addition was created between the human proteins physically associated with SARS-CoV-2 and their first neighboring proteins of the immune system. This network was generated using the human proteome of the Cytoscape StringApp (Szklarczyk et al., 2015; Doncheva et al., 2019), which imports protein-protein interaction data from the STRING database (Szklarczyk et al., 2015). The degree centrality represents the number of edges the node has in a network (López-Cortés et al., 2018; López-Cortés et al., 2020b), and it was calculated using the CytoNCA app (Tang et al., 2015). All nodes and edges were organized through the organic layout, which produces clear representations of complex networks, and lastly, the immune system PPi network was visualized through the Cytoscape software v.3.7.1 (Shannon et al., 2003).

Interestingly, Overmyer *et al.* published a large-scale multi-omic analysis identifying 146 significantly expressed proteins in patients with severe COVID-19 (Overmyer et al., 2020). We located these proteins in our immune system PPi network and generated the immune system PPi subnetwork encompassing the significantly expressed proteins in severe COVID-19 and their first neighbor nodes (cutoff = 0.9). Subsequently, we ranked the overexpressed and underexpressed proteins according to the highest degree centrality.

Additionally, Bouhaddou *et al.* published the global phosphorylation landscape of SARS-CoV-2 infection identified 97 significantly expressed proteins in Vero E6 cells (Bouhaddou et al., 2020). We located these proteins in both networks and ranked the phosphorylated proteins according to the highest degree centrality. Lastly, human proteins physically associated with the SARS-CoV-2 proteins, immune system proteins, significantly expressed proteins in severe COVID-19, and significantly expressed phosphorylated proteins in SARS-CoV-2 infection in Vero E6 cells were differentiated by colors in both the immune system PPi network and subnetwork.

### Functional Enrichment Analysis

The functional enrichment analysis gives curated signatures of protein sets generated from omics-scale experiments (Reimand et al., 2019). We performed the enrichment analysis to validate the correlation between the immune system PPi subnetwork and biological annotations related to severe COVID-19, using the protein set of the immune system PPi network as background set. The enrichment was calculated using g:Profiler version e101_eg48_p14_baf17f0 (https://biit.cs.ut.ee/gprofiler/gost) to obtain significant annotations (Benjamini-Hochberg false discovery rate - FDR < 0.001) related to GO: biological processes, the Kyoto Encyclopedia of Genes and Genomes (KEGG) signaling pathways, and Reactome signaling pathways (Wang et al., 2016; Slenter et al., 2018; Raudvere et al., 2019; Jassal et al., 2020). Lastly, the enrichment analysis was visualized in a Manhattan plot, and the significant terms related to the immunopathology of severe COVID-19 were manually curated.

### Single-Cell RNA Sequencing Data

Ziegler *et al.* analyzed human scRNA-seq data to uncover potential targets of SARS-CoV-2 amongst tissue-resident cell subsets. They discovered *ACE2* and *TMPRSS2* co-expressing in goblet secretory cells from nasal passages, type II pneumocytes from lung epithelial cells, and absorptive enterocytes from ileal epithelial cells (Ziegler et al., 2020).

After constructing the immune system PPi network between the human proteins physically associated with the SARS-CoV-2 proteins, immune system proteins, and significantly expressed proteins in severe COVID-19, we compared the transcriptomics data of the network nodes between 10 nasal passage cells (goblet cell, basal cell of olfactory epithelium, ciliated cell, endothelial cell, fibroblast cell, glandular epithelial cell, mast cell, myeloid cell, plasma cell, and T cell), 15 lung epithelial cells (ciliated cell, lymphatic cell, fibroblast 1, fibroblast 2, macrophage 1, macrophage 2, macrophage 3, mast cell, monocytes 1, monocytes 2, neutrophil cell, proliferating cell, T cell, type I pneumocytes, and type II pneumocytes), and 9 ileal epithelial cells (cycling stem cell, early enterocyte 1, early enterocyte 2, absorptive enterocyte, enteroendocrine cell, goblet cell, quiescent stem cell, TA G1S cell, and TA G2M cell) to identify significantly expressed genes in goblet secretory cells, type II pneumocytes, and absorptive enterocytes.

The transcriptomics data was taken from the ‘COVID-19 Studies’ section of the Single Cell Portal (https://singlecell.broadinstitute.org/single_cell/covid19), and the Alexandria Project (https://alexandria-scrna-data-library.readthedocs.io/en/latest/introduction.html). The three single-cell databases analyzed were: 1) nasal passage cells (Ordovas-Montanes et al., 2018) (https://singlecell.broadinstitute.org/single_cell/study/SCP253/allergic-inflammatory-memory-in-human-respiratory-epithelial-progenitor-cells#study-visualize), 2) lung epithelial cells (Ziegler et al., 2020) (https://singlecell.broadinstitute.org/single_cell/study/SCP814/human-lung-hiv-tb-co-infection-ace2-cells#study-visualize), and 3) ileal epithelial cells (Fujii et al., 2018) (https://singlecell.broadinstitute.org/single_cell/study/SCP817/comparison-of-ace2-and-tmprss2-expression-in-human-duodenal-and-ileal-tissue-and-organoid-derived-epithelial-cells#study-visualize). Lastly, it is important to clarify that the scRNA-seq analyses were done in cells non exposed to the novel coronavirus.

The criteria of analysis of transcriptomics data of nasal passage cells, lung epithelial cells, and ileal epithelial cells was the following: ‘t-distributed stochastic neighbor embedding (t-SNE) cell types’ as load cluster, ‘cell type ontology label’ as selected annotation, and ‘all cells’ as subsampling threshold. Additionally, we adjust the mRNA expression taking into account the Z-scores, that is, overexpressed mRNAs with Z-scores > 2 and underexpressed mRNAs with Z-scores < −2. Regarding visualization of transcriptomics data, we designed heatmaps to compare the expression between cell types, dot plots to visualize the percentage of cells expressing, box plots to compare the expression scores of multiple genes for each cell type taking into account the mean log normalized expression, and 2D t-SNE to visualize the expression score of significantly expressed multiple genes per subpopulation cell.

### Drug Repurposing

After identifying the significantly expressed biological molecules present in the scRNA-seq analyses of *ACE2* and *TMPRSS2* co-expressing human cells, we evaluated the druggability of these molecules, and subsequently perform the drug repurposing analysis.

From all the 75 previously identified and significantly expressed biological molecules, only 31 had identification number in the ChEMBL database (https://www.ebi.ac.uk/chembl) (Gaulton et al., 2017), and from these 31 proteins, all compounds were extracted from ChEMBL as follow: 1) all reported interactions with (IC50, Ki, EC50, and GI50) where extracted from ChEMBL version 26; 2) all extracted interactions were labeled as active (1) or inactive (0) if values are less than 10 μM; and 3) if more than one report (active or inactive) is available for the same compound-target interaction, the final criteria (active or inactive) was assigned considering the 75% of the information or rejected otherwise. From the 31 proteins, only 25 had identified molecules with active/inactive interactions after considering the previous filters. Hence, we identified 25 potential therapeutic targets for drug repurposing against COVID-19.

DeepChem package and Python Jupyter Notebooks (Oliver, 2013) were used to predict if drugs (DrugBank compounds) could be active for multiple protein targets (Oliver, 2013) (https://github.com/deepchem/deepchem). DrugBank (https://www.drugbank.ca/) contains comprehensive information about drugs, their mechanism of action, and their targets (Wishart et al., 2006; Wishart et al., 2018). The calculations used the GPU of Google Colab and the correspondent scripts could be found at GitHub repository: https://github.com/muntisa/immuno-drug-repurposing-COVID-19. The fully-connected deep neuronal networks (FCNNs) have been used to find the best multitask classification model using 1,024 molecular circular fingerprints (CFPs) as input descriptors for 15,377 ChEMBL compounds and activity (1/0) for the 25 therapeutic targets as outputs/tasks (Wu et al., 2018). The best model resulted from a grid search for the best parameters have been used to predict the activity of 10,672 drugs for the 25 targets. The performance of the classifiers used during the training, grid search and test evaluation of the best model was the area under curve (AUC) of the receiver operating characteristic (ROC) curve (AUROC) (Hastie et al., 2009), the default metric in DeepChem package. The ROC curve is defined by the True Positive Rate (TPR) (or Sensitivity) vs. the False Positive Rate (FPR) (or 1-Specificity) for each of the class of the multi-task classifier for different class probability thresholds. TPR = TP/(TP + FN), FPR = FP/(FP + TN), where TP = True Positive; FP = False Positive; TN = True Negative; FN = False Negative (from the confusion matrix that summarizes the results of testing the classifier). AUROC represents the area under the ROC curve, with values between 0 and 1 (1 = perfect model; 0.5 = no skill/random model).

The main script of the repository (Immuno-Drug-Repurposing-DeepChem-MultitaskClassification.ipynb) is presenting all the methodology with python code and results. The repository folder “datasets” contains the dataset with the ChEMBL ID, SMILES formula, and the class of protein target (multiclass_origDS_noDB.csv). The dataset that will be used by the classifier contains the SMILES formulas of 15,377 ChEMBL compounds that interacts with 25 different protein targets with the following UniProt IDs: O00571, P00533, P01024, P01130, P04233, P07339, P08962, P09668, P11021, P15291, P16070, P17301, P21741, P25774, P25963, P26006, P27361, P35222, P40763, P50591, P55085, Q15904, Q16665, Q99519, and Q99814. This means that the dataset was composed by 15,377 examples with 25 classes. The multi-task classification model will be able to predict if a compound with a SMILE formula could have one or more protein targets simultaneously, using separated tasks/outputs for each of the 25 proteins. It is not a simple classification with only an output (class) that can predict only a protein value from the 25 possible targets). The prediction molecules that will be evaluated with the best classifier can be found in DB_toPredict.csv (DataBank ID, SMILES formulas, and the classes to predict). The input SMILES formulas will be used to calculate molecular descriptors for all molecules (as model inputs).

In the first step, CFPs molecular descriptor have been calculated for both ChEMBL dataset and DrugBank prediction set (Gaulton et al., 2017; Wishart et al., 2018) as a vector of 1,024 values for each compound. Thus, the dataset to build the future classifier has 1,024 input features in 15,377 examples with 25 output classes (protein target).

In order to build a classifier (model), the training of the model should be done with a training subset and the final model should be tested for performance with a test subset that was not used during the training process. In addition, if different classifiers with different parameters are used during the training, there is a need of an extra validation subset to decide the best classifier (model) using a specific metrics (in our scripts: AUROC). Thus, the dataset was splitted into 80%-10%-10% training-validation-test subsets using RandomStratifiedSplitter (to maintain the same ratio between the examples in all 25 classes as in the initial dataset). The training and validation subsets were used to find the best hyperparameters for the FCNN with 1,000 neurons (MultitaskClassifier from DeepChem package). The constant parameters are activation functions as relu, momentum of 0.9, weights initialization using Glorot uniform method (Xavier uniform initializer), learning rate of 1e-3, decay of 1e-6, 1o epochs, a single hidden layer (additional parameters could be found in the main notebook of the repository). During the grid search for the best model, 64 classifiers have been optimized with different combination of the following parameters: batch size = (128, 515), dropouts = (0.0, 0.1, 0.2, 0.3), batch normalization = (False, True), and hidden layer sizes (number of neurons) = (100, 500, 1,000, 1,024). Thus, the training subset was used for training of each model/classifier and the validation subset was used to decide the best model.

The test set was used to verify the performance of the best model for each task/protein target (see Supplementary Table S2). AUROC for the test subset was between 0.935 and 1.000 (mean AUROC = 0.989; standard deviation (SD) = 0.019). Additional results such as the AUROC values for training, validation and test subset for each protein target (task/class) are presented into the folder “results” as multitasks_metrics_best.csv.

The best model has 1,000 neurons in a hidden layer (dropout of 0.5) with all parameters as 'activation': 'relu', 'momentum': 0.9, 'batch_size': 124, 'init': 'glorot_uniform', 'data_shape': (1024), 'learning_rate': 0.001, 'decay': 1e-06, 'nb_epoch': 1, 'nesterov': False, 'dropouts': (0.5), 'nb_layers': 1, 'batchnorm': False, 'layer_sizes': (1000), 'weight_init_stddevs': (0.1), 'bias_init_consts': (1.0), 'penalty': 0.0. This classifier was used to predict the activity of 10,672 drugs from DataBank for the 25 immune system targets: DDX3X, EGFR, C3, LDLR, CD74, CTSD, CD63, CTSH, HSPA5, B4GALT1, CD44, ITGA2, MDK, CTSS, NFKBIA, ITGA3, MAPK3, CTNNB1, STAT3, TNFSF10, F2RL1, ATP6AP1, HIF1A, NEU1, and EPAS1 (see Supplementary Table S7 and multitasks_predictions_best.csv in repository folder “results”). Lastly, the best predicted drug-target associations were evaluated according to its first ATC level (https://www.whocc.no/atc_ddd_index/), drug category, mechanism of action, approval status by the US Food & Drug Administration (FDA) or the European Medicines Agency (EMA), the pharmacological indications, and the current involvement in COVID-19 clinical trials (https://www.clinicaltrials.gov/ct2/results?cond=COVID-19).

## Results

### Immune System Protein-Protein Interactome Network

In biological systems, specialized pathogens (i.e., SARS-CoV-2) employ a suite of virulent proteins, which interact with key targets in host interactomes to extensively rewire the flow of information and cause diseases, such as COVID-19 (Vidal et al., 2011; Pan et al., 2016; Kumar et al., 2020). The human proteins physically associated with SARS-CoV-2 are the first line of host proteins, which also interacts with molecular components involved in a wide spectrum of biological processes and signaling pathways within the cell. Therefore, analyzing the interactome of immune system proteins may reveal novel components in SARS-CoV-2 immunopathogenesis.

**FIGURE 2:**
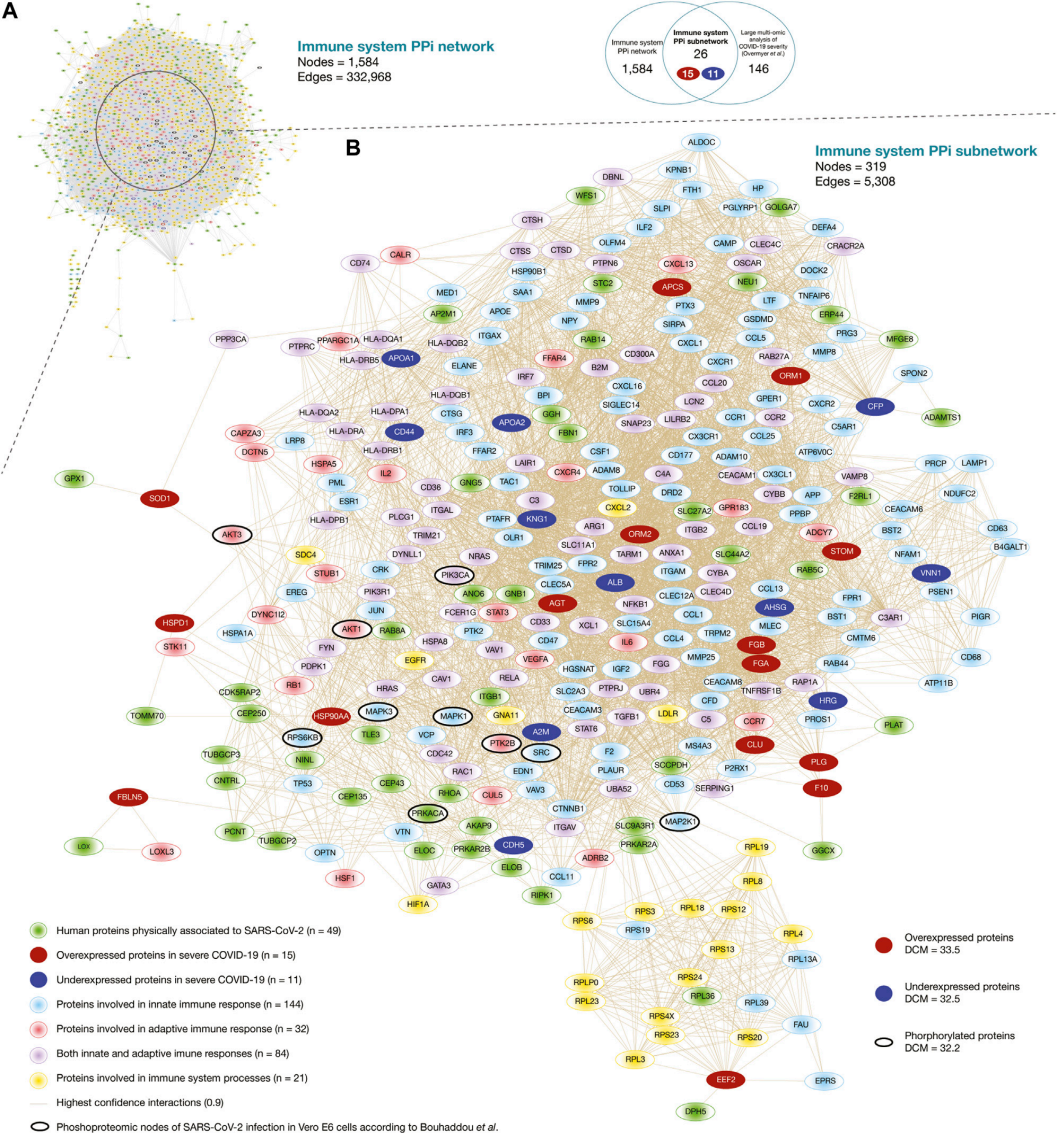
Immune system protein interactomes. **(A)** Immune system PPi network. **(B)** Immune system PPi subnetwork made up of human proteins physically associated with SARS-CoV-2 proteins, immune system proteins, significantly expressed proteins in severe COVID-19, and significantly expressed proteins of SARS-CoV-2 infection in Vero E6 cells. DCM: degree centrality mean.

Here, we generated the immune system PPi network encompassing 1,584 nodes and 332,968 edges (Figure 2A). Of them, 256 human proteins physically associated with SARS-CoV-2 proteins had high-confidence interactions (cutoff = 0.9) with 1,390 immune system proteins belonging to the first neighbor nodes (Supplementary Table S3). The degree centrality mean of the human proteins physically associated with SARS-CoV-2 proteins was 23.6, and proteins with the highest degree centrality were GNB1, GNG5, RBX1, RHOA, and TCEB1. On the other hand, the degree centrality mean of the immune system protein was 44.5, and proteins with the highest degree centrality were UBA52, APP, FPR2, NCBP1, and NCBP2. Additionally, we have identified 40 significantly expressed phosphorylated proteins of SARS-CoV-2 infection according to the global phosphorylation landscape in Vero E6 cells published by Bouhaddou et al. (2020). The degree centrality mean of the phosphorylated proteins was 59.8, and proteins with the highest degree centrality were PIK3CA, MAPK1, MAPK3, SRC, and AKT1 (Supplementary Table S4). Lastly, Supplementary Figure S1 details an expanded visualization of the immune system PPi network.

Figure 2B shows the immune system PPi subnetwork encompassing 319 nodes and 5,308 edges. Of them, 26 significantly expressed proteins in severe COVID-19 (15 overexpressed and 11 underexpressed) (Overmyer et al., 2020) had high-confidence interactions (cutoff = 0.9) with 49 human proteins physically associated with SARS-CoV-2 proteins, and with 281 immune system proteins belonging to the first neighbor nodes. The degree centrality mean of the overexpressed proteins was 33.5, and proteins with the highest degree centrality were STOM, HSP90AA1, AGT, ORM1, and ORM2. On the other hand, the degree centrality mean of the underexpressed protein was 32.5, and proteins with the highest degree centrality were KNG1, CFP, ALB, AHSG, and APOA1. Additionally, we have identified 10 significantly expressed phosphorylated proteins of SARS-CoV-2 infection in Vero E6 cells in our subnetwork. The degree centrality mean of the phosphorylated proteins was 32.2, and proteins with the highest degree centrality were PIK3CA, MAPK1, SRC, MAPK3, and AKT1 (Supplementary Table S4). Although it has been shown that hubs of high-degree nodes are targets of numerous human viral (Calderwood et al., 2007; De Chassey et al., 2008; Gulbahce et al., 2012; Pan et al., 2016; Huttlin et al., 2017), and are highly correlated with pathogenicity in cancer (López-Cortés et al., 2018; López-Cortés et al., 2020b; Cabrera-andrade, 2020), COVID-19 is a novel disease and requires more in-depth studies.

### Functional Enrichment Analysis

The functional enrichment analysis was performed to validate the correlation between the immune system PPi subnetwork and biological annotations related to severe COVID-19. Therefore, after generating the subnetwork encompassing 319 immune system proteins, we performed a functional enrichment analysis using g:Profiler to obtain significant annotations (Benjamini-Hochberg FDR < 0.001) related to GO: biological processes, KEGG signaling pathways, and Reactome signaling pathways (Wang et al., 2016; Slenter et al., 2018; Raudvere et al., 2019; Jassal et al., 2020).

**FIGURE 3:**
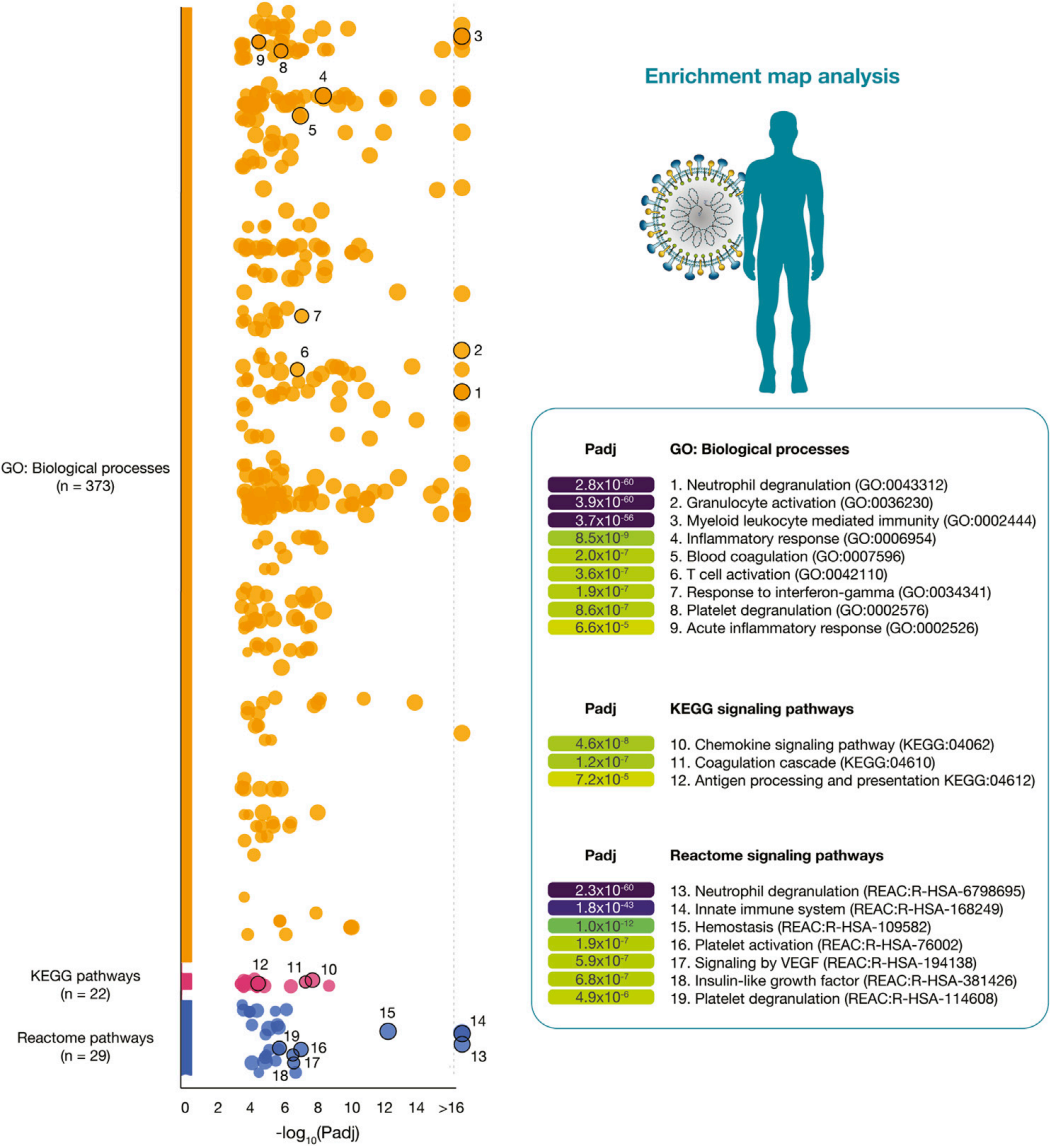
Enrichment map analysis of the immune system PPi subnetwork. Significant GO: biological processes, KEGG signaling pathways, and Reactome signaling pathways.

Figure 3 details a Manhattan plot of 373 GO: biological processes, 22 KEGG signaling pathways, and 29 Reactome signaling pathways significantly associated with the 319 immune system proteins. However, after a manual curation of GO terms related to the immunopathology of severe COVID-19, the most significant GO: biological processes were neutrophil degranulation (2.8 × 10^−60^), granulocyte activation (3.9 × 10^−60^), myeloid leukocyte mediated immunity (3.7 × 10^−56^), inflammatory response (8.5 × 10^−9^), blood coagulation (2.0 × 10^−7^), T-cell activation (3.6 × 10^−7^), response to interferon-gamma (1.9 × 10^−7^), platelet degranulation (8.6 × 10^−7^), and acute inflammatory response (6.6 × 10^−5^). The most significant KEGG signaling pathways related to severe COVID-19 were chemokine signaling pathway (4.6 × 10^−8^), coagulation cascade (1.2 × 10^−7^), and antigen presentation (7.2 × 10^−5^). Lastly, the most significant Reactome signaling pathways related to severe COVID-19 were neutrophil degranulation (2.3 × 10^−60^), innate immune system (1.8 × 10^−43^), hemostasis (1.0 × 10^−12^), signaling by VEGF (5.9 × 10^−7^), insulin-like growth factor (6.8 × 10^−7^), and platelet degranulation (4.9 × 10^−6^) (Supplementary Table S5).

### Single-Cell RNA Sequencing Data Analysis

Omics medicine has evolved the way for identifying therapeutically actionable targets for complex diseases. However, one of the major limitations is the gene expression variability due to the cellular heterogeneity of organs (Gawel et al., 2019). Single-cell biology is a powerful approach that provides unprecedented resolution to the cellular and molecular underpinnings of biological processes and signaling pathways of diseases in order to find therapeutic targets (Ballestar et al., 2020). For instance, the significant overexpression of programmed death 1 (PD-1) in innate lymphoid cells as therapeutic target for cancer immunotherapy (Yu et al., 2016).

Regarding COVID-19, there are several single-cell studies focused on understanding the transcriptional and proteomics insights into the host response for drug discovery (Ballestar et al., 2020; Yang X et al., 2020; Di Giorgio et al., 2020; Wu M et al., 2020; Park and Lee, 2020; Prokop et al., 2020). Ziegler *et al.* discovered *ACE2* and *TMPRSS2* co-expressing cells in nasal goblet secretory cells, lung type II pneumocytes, and ileal absorptive enterocytes through scRNA-seq data analyses (Ziegler et al., 2020). Once we delimited the interactions between human proteins physically associated with SARS-CoV-2, and immune system proteins (immune system PPi network), we analyzed the transcriptomics data of the 1,584 nodes using three single-cell databases incorporated into the ‘COVID-19 Studies’ section of the Alexandria Project (see Methods), in order to reveal potential therapeutic targets for drug repurposing against COVID-19.

**FIGURE 4:**
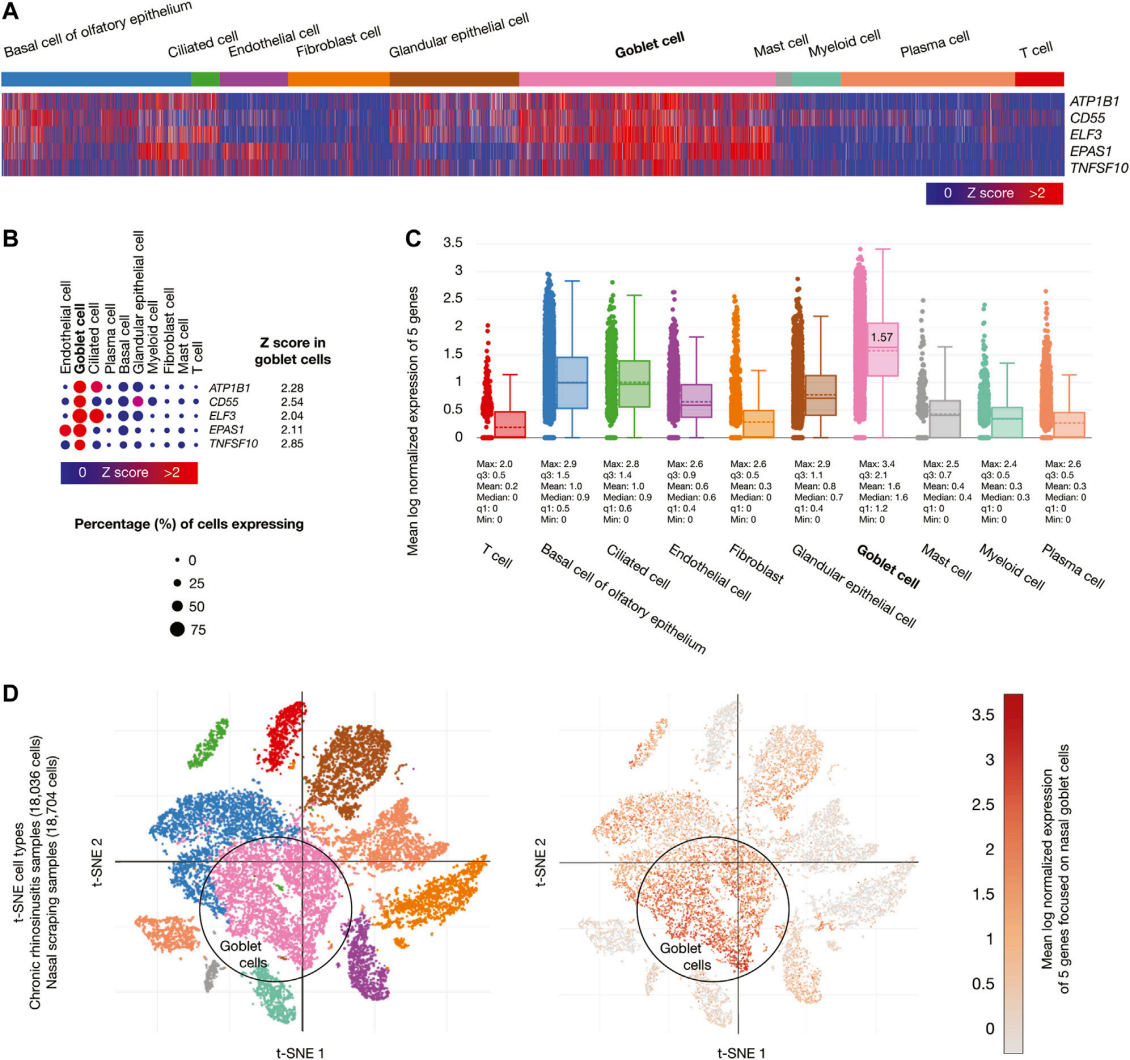
Single-cell RNA-sequencing data analysis of the high-confidence immune system nodes in nasal passage cells. **(A)** Heatmap of significant overexpressed genes (Z-score > 2) in nasal goblet secretory cells. **(B)** Dot plot of significant overexpressed genes in nasal goblet secretory cells and percentage of cells expressing. **(C)** Box plots of nasal passage cell types according to their mean log normalized expression. **(D)** t-distributed stochastic neighbor embedding cell type and mean log normalized expression focused on nasal goblet secretory cells.

Chronic rhinosinusitis samples (18,036 cells) developed by allergic inflammation, and nasal scraping samples (18,704 cells) conform the nasal passage cells. Figure 4A shows a heatmap of the five genes whose mRNAs were significantly overexpressed (Z-score > 2) in goblet cells. Figure 4B shows a dot plot detailing the five overexpressed genes, its Z-scores between 2.04 and 2.85, and the percentage of goblet cells expressing the overexpressed genes (>50%). Figure 4C shows box plots comparing the mean log normalized expression of the five overexpressed genes in nasal passage cells. Goblet cells had the highest mean log normalized expression (1.57) compared to the other cells. Figure 4D projected the expression scores of the significantly expressed multiple genes (n = 5) onto 2D t-SNEs per subpopulation cell (total = 10 subpopulation cells). In summary, five immune system genes were overexpressed in the goblet cells from nasal passages.

**FIGURE 5:**
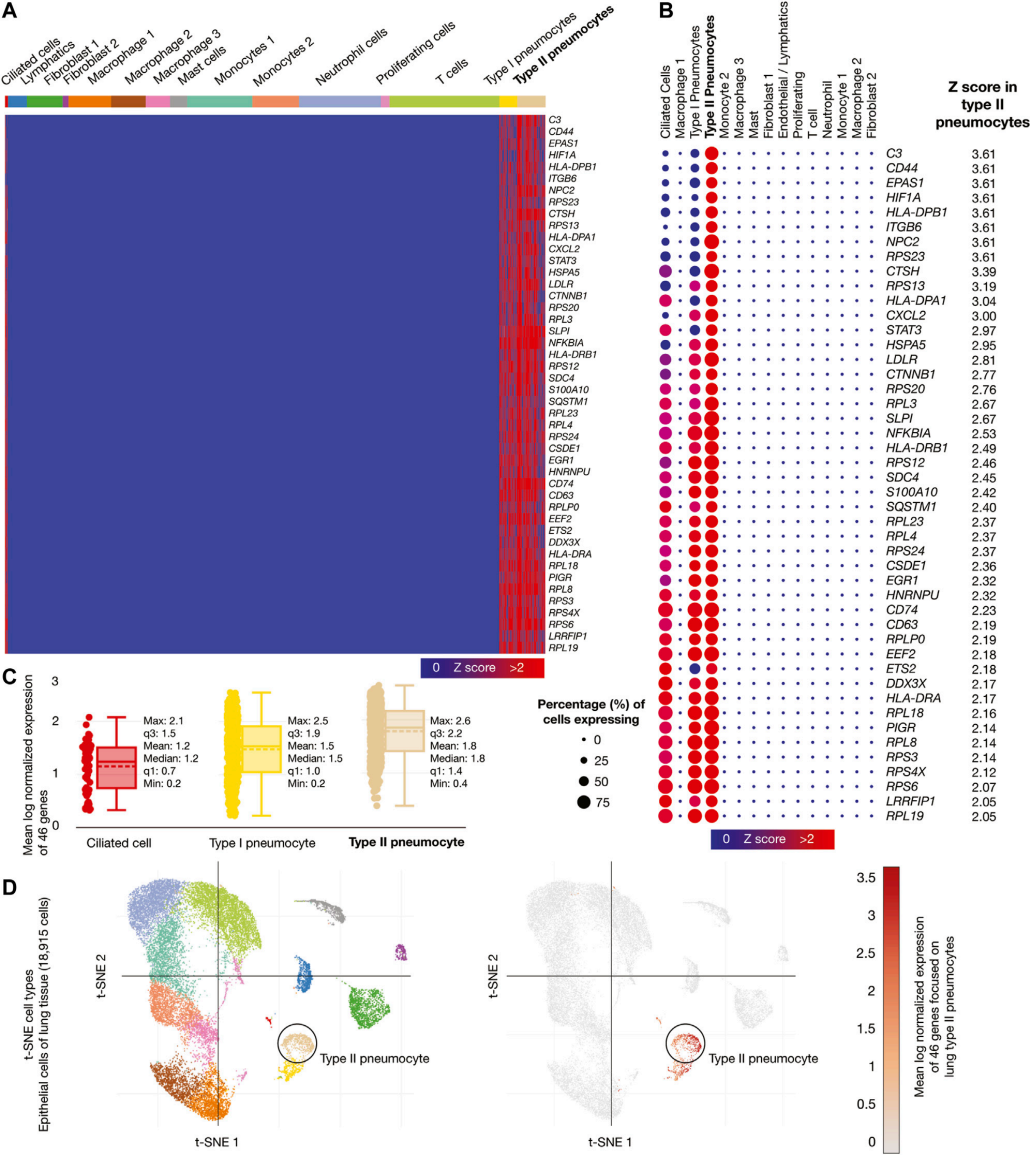
Single-cell RNA-sequencing data analysis of the high-confidence immune system nodes in lung cells. **(A)** Heatmap of significant overexpressed genes (Z-score > 2) in lung type II pneumocytes. **(B)** Dot plot of significant overexpressed genes in lung type II pneumocytes and percentage of cells expressing. **(C)** Box plots of lung cell types according to their mean log normalized expression. **(D)** t-distributed stochastic neighbor embedding cell type and mean log normalized expression focused on lung type II pneumocytes.

Epithelial cells of lung tissue (18,915 cells) were the second single-cell database analyzed. Figure 5A shows a heatmap of the 46 genes whose mRNAs were significantly overexpressed in lung type II pneumocytes. Figure 5B shows a dot plot detailing the 46 overexpressed genes, its Z-scores between 2.05 and 3.61, and the percentage of type II pneumocytes expressing the overexpressed genes (>50%). Figure 5C shows box plots comparing the mean log normalized expression of the 46 overexpressed genes in lung cells. Type II pneumocytes had the highest mean log normalized expression (1.78) compared to other cells. Figure 5D projected the expression scores of the significantly expressed multiple genes (n = 46) onto 2D t-SNEs per subpopulation cell (total = 15 subpopulation cells). In summary, 46 immune system genes were overexpressed in type II pneumocytes from lung cells.

**FIGURE 6:**
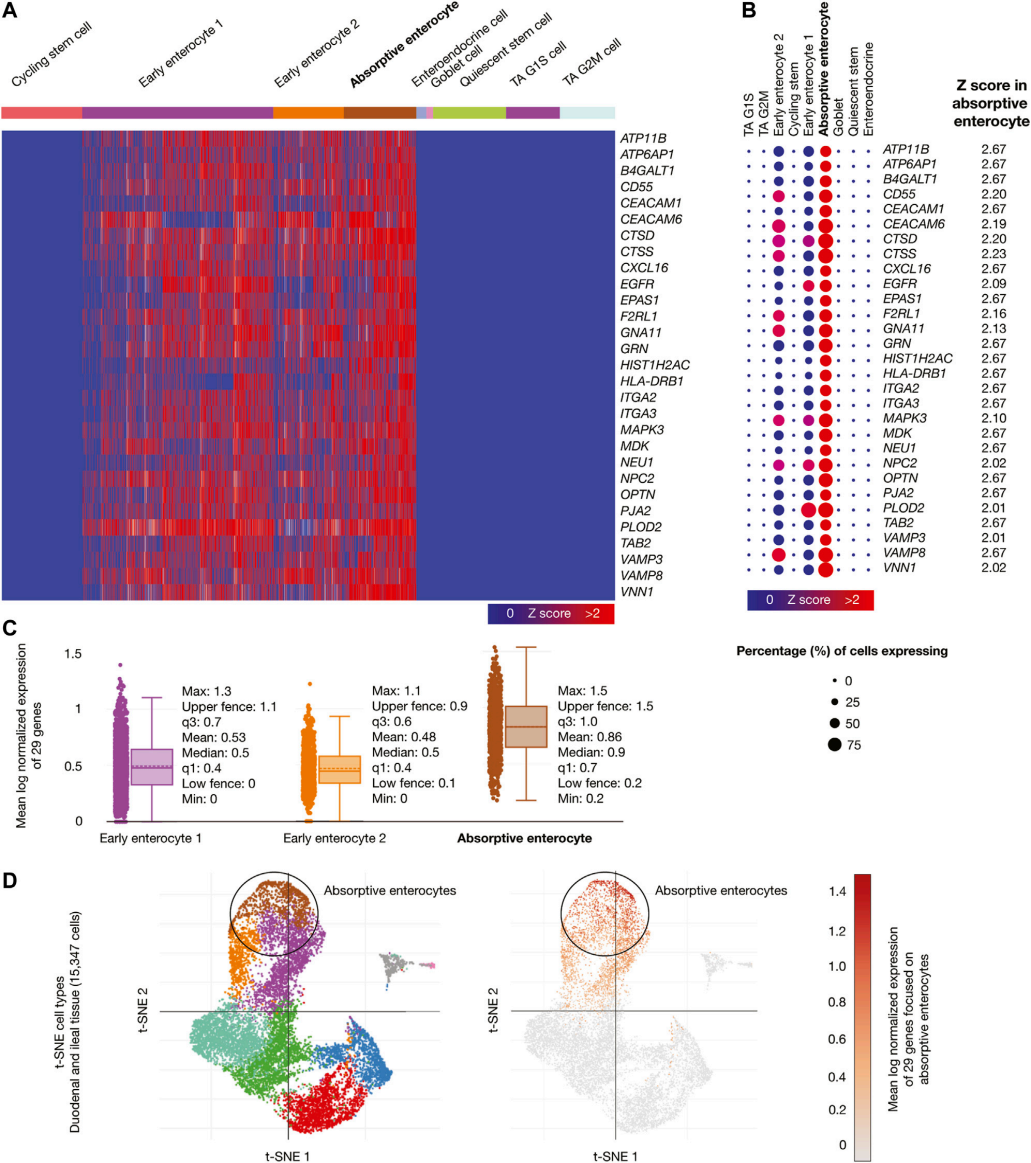
Single-cell RNA-sequencing data analysis of the high-confidence immune system nodes in intestine cells. **(A)** Heatmap of significant overexpressed genes (Z-score > 2) in ileal absorptive enterocytes. **(B)** Dot plot of significant overexpressed genes in ileal absorptive enterocytes and percentage of cells expressing. **(C)** Box plots of intestine cell types according to their mean log normalized expression. **(D)** t-distributed stochastic neighbor embedding cell type and mean log normalized expression focused on ileal absorptive enterocytes.

Samples from adult human duodenum and ileum (15,347 cells) were the third single-cell database analyzed. Figure 6A shows a heatmap of genes whose mRNAs were significantly overexpressed in ileal absorptive enterocytes. Figure 6B shows a dot plot detailing the 29 overexpressed genes, its Z-scores between 2.02 and 2.67, and the percentage of ileal absorptive enterocytes expressing the overexpressed genes (>50%). Figure 6C shows box plots comparing the mean log normalized expression of the 29 overexpressed genes in ileal epithelial cells. Absorptive enterocytes had the highest mean log normalized expression (0.86) compared to other cells. Figure 6D projected the expression scores of the significantly expressed multiple genes (n = 29) onto 2D t-SNEs per subpopulation cell (total = 9 subpopulation cells). In summary, 29 immune system genes were overexpressed in absorptive enterocytes from ileal epithelial cells. The biological function of the 75 overexpressed genes is fully detailed in Supplementary Table S6).

### Drug Repurposing

**FIGURE 7:**
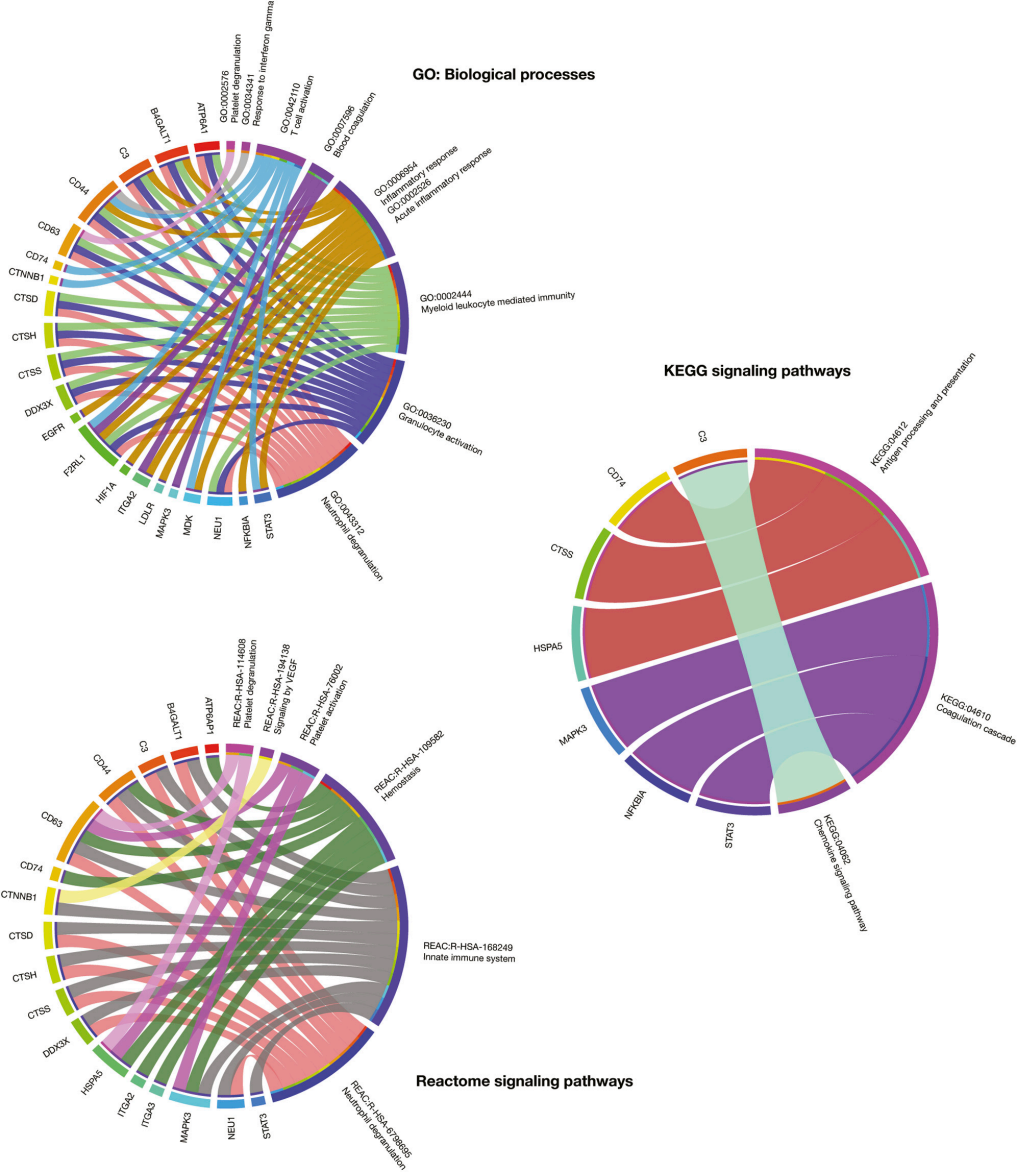
Circos plots that detail association between GO: biological processes, KEGG signaling pathways, and Reactome signaling pathways and the most relevant immune system proteins for drug repurposing.

The current work proposes an innovative virtual high-throughput screening to predict the activity of 10,672 compounds for 25 immune system targets fully detailed in the Supplementary Table S7. The other 50 targets had not identified molecules with active/inactive interactions in the ChEMBL database as previously explained in Methods section. Interestingly, the 25 potential therapeutic targets analyzed not only were relevant in the immune system PPi subnetwork and the scRNA-seq analyses, but also had significant associations with biological processes and signaling pathways relevant to severe COVID-19 (Overmyer et al., 2020). For instance, ATP6A1, B4GALT1, C3, CD44, CD63, CTSD, CTSH, CTSS, DDX3X, F2RL1, and NEU1 were involved in neutrophil degranulation; F2RL1, ITGA2, MAPK3, NFKBIA, and STAT3 in blood coagulation or coagulation cascade; ATP6AP1, CD44, CD63, CD74, HSPA5, ITGA2, ITGA3, and MAPK3 in hemostasis; lastly, CD63, HSPA5, and MAPK3 in platelet degranulation (Figure 7).

The classification model was based on the molecular Circular Fingerprints descriptors (calculated using SMILES formulas) of 15,377 ChEMBL compounds and its 25 therapeutic targets as outputs/tasks. The best model was obtained after a hyperparameter grid search (64 topologies) as a fully connected deep neuronal networks with 1,000 neurons in one hidden layer, with the mean AUROC of 0.989 ± 0.019 (AUROC between 0.935 and 1.000 for 25 classes). Our free GitHub repository contains the Jupyter notebook as python script using DeepChem methodology, datasets, calculated descriptors, best model, metrics of the model, and predictions. After applying the best classification model, we evaluated drugs taking into account the first ATC levels associated to COVID-19 symptoms, drug category, mechanism of action, pharmacological indications, and the best ranked AUROC values (threshold > 0.8). Consequently, on one hand, we obtained 44 approved drugs, 16 compounds under investigation, and 35 experimental compounds with the highest affinities for 15 immune system proteins (Supplementary Table S8). On the other hand, we obtained four approved drugs, nine compounds under investigation, and 16 experimental compounds with the highest multi-target affinities for nine immune system proteins (Supplementary Table S9).

**FIGURE 8:**
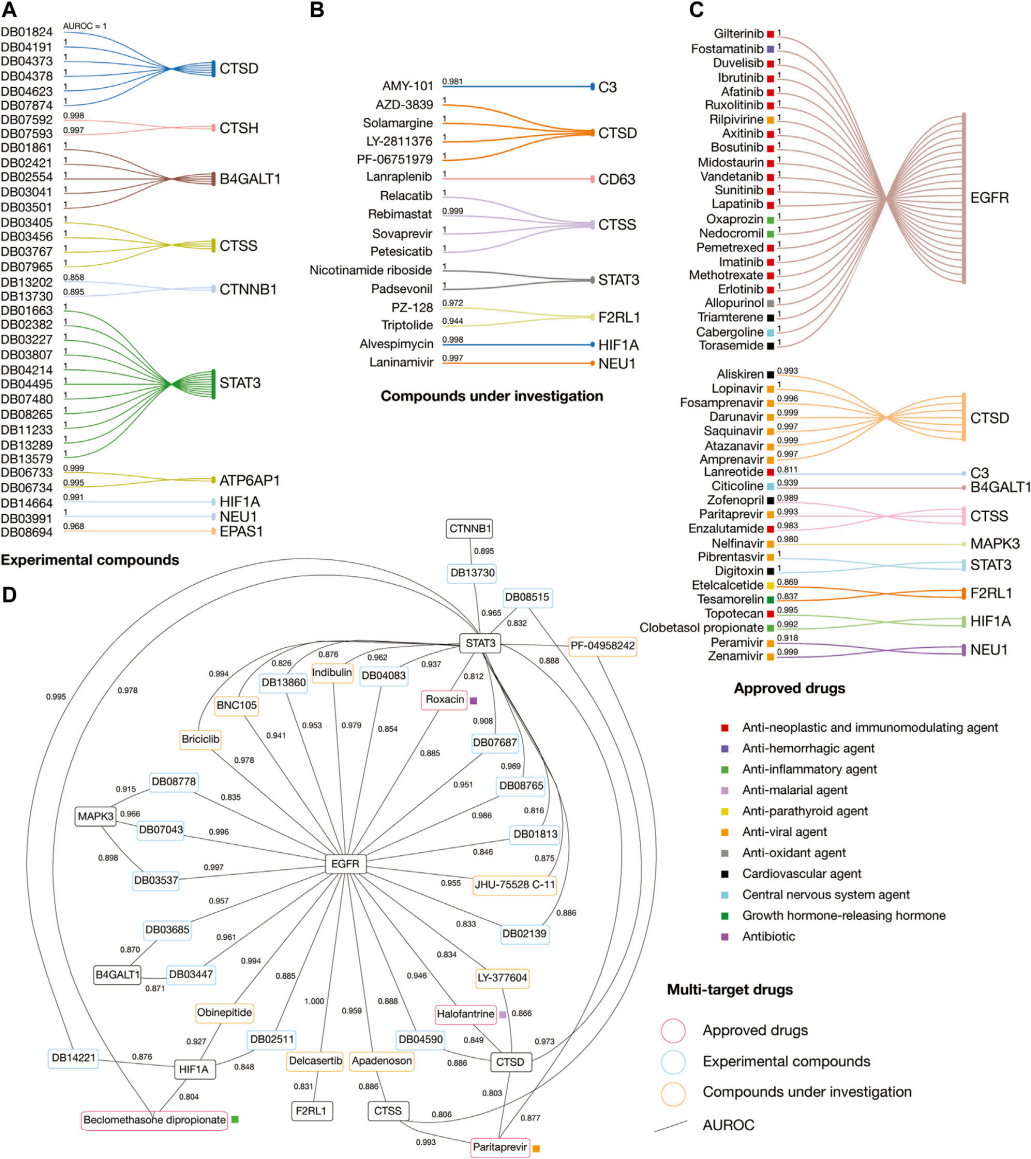
Drug repurposing analyses applying artificial neural networks. **(A)** Best-predicted experimental compounds per immune system protein target. **(B)** Best-predicted compounds under investigation per immune system protein target. **(C)** Best-predicted approved drugs per immune system protein target. **(D)** Best-predicted multi-target experimental compounds, compounds under investigation, and approved drugs. AUROC: Area under the receiver operating characteristic.

Figure 8 details the AUROC affinity score of the best-predicted experimental compounds, compounds under investigation, and approved drugs per immune system protein target and multi-targets. We found eleven different categories of approved drugs, the anti-neoplastic and immunomodulating agents were lanreotide, enzalutamide, topotecan, erlotinib, methotrexate, imatinib, pemetrexel, lapatinib, sunitinib, vandetanib, midostaurin, bosutinib, axitinib, ruxolitinib, afatinib, ibrutinib, duvelisib, and gilterintinib; the anti-hemorrhagic agent was fostamatinib; the anti-inflammatory agents were clobetasol propionate, nedocromil, oxaprozin, and beclomethasone dipropionate; the anti-malarial agent was halofantrine; the anti-parathyroid agent was etelcalcetide; the anti-viral agents were amprenavir, atazanavir, saquinavir, darunavir, fosamprenavir, lopinavir, paritrapevir, nelfinavir, pibrentasvir, zanamivir, peramivir, and rilpivirine; the antioxidant agent was allopurinol; the cardiovascular agents were aliskiren, zofenopril, digitoxin, torasemide, and triamterene; the central nervous system agents were citicoline and cabergoline; the growth hormone-releasing hormone was tesamorelin; and the only antibiotic was rosoxacin.

**FIGURE 9:**
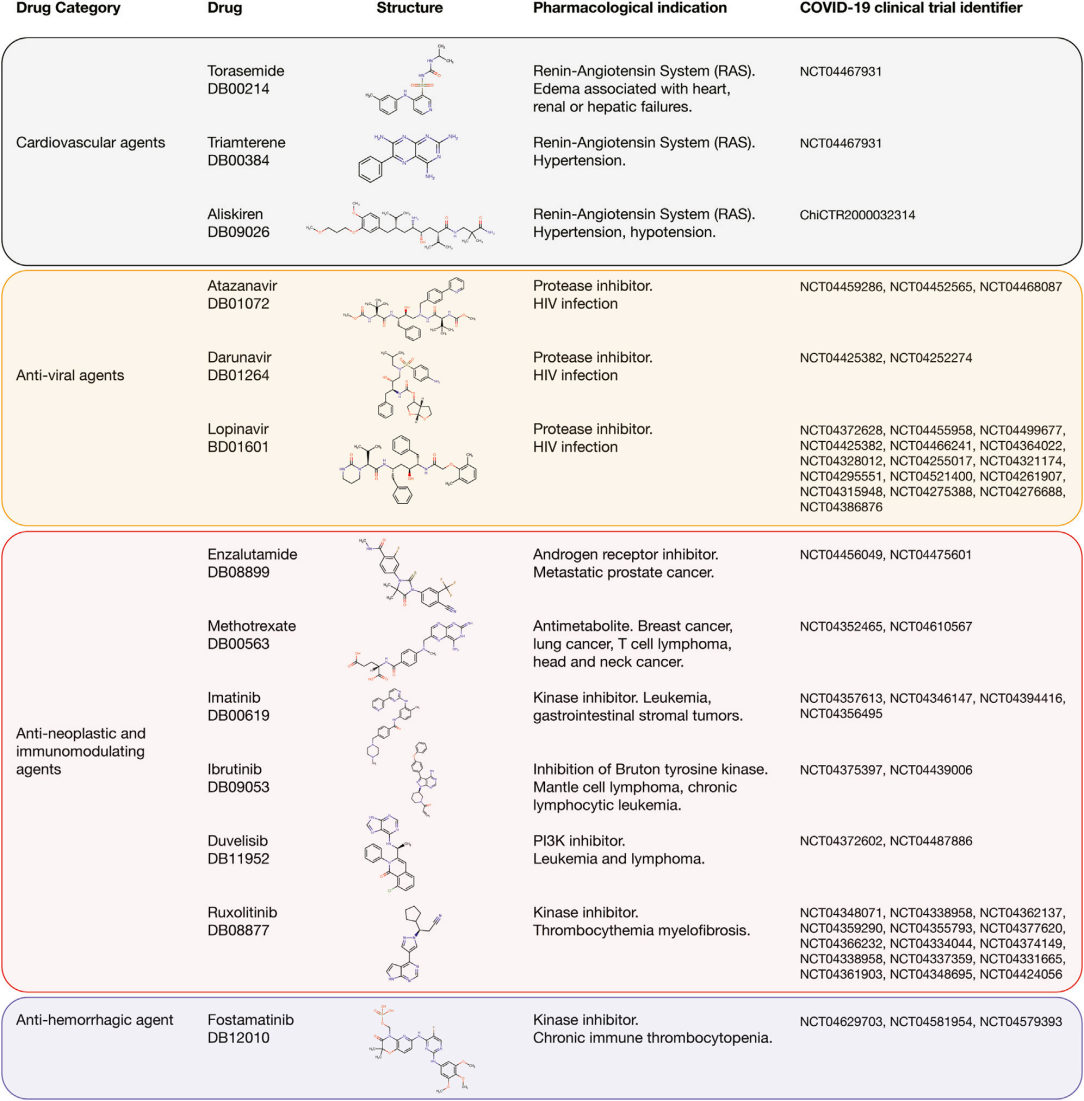
Best-predicted approved drugs involved in COVID-19 clinical trials. Cardiovascular agents, anti-viral agents, anti-neoplastic and immunomodulating agents, and anti-hemorrhagic agent with their respective clinical trial identifier number, pharmacological indication, and chemical structure according to DrugBank.

Interestingly, 13 (27%) of the 48 best-predicted approved drugs are currently involved in approximately 54 COVID-19 clinical trials as detailed in Figure 9. The cardiovascular agents with clinical trials are aliskiren, torasemide, and triamterene. Aliskiren had an AUROC affinity of 0.993 on CTSD, and it is a renin inhibitor used to treat hypertension; torasemide had an AUROC affinity of 1.0 on EGFR, and it is used to treat edema associated with heart, renal, and hepatic failures; and triamterene had an AUROC affinity of 1.0 on EGFR, and it is used to treat hypertension. The anti-viral agents with clinical trials are atazanavir, darunavir, and lopinavir. Atazanavir had an AUROC affinity of 0.997 on CTSD; darunavir had an AUROC affinity of 0.999 on CTSD, and lopinavir had an AUROC affinity of 1.0 on CTSD. All of them are protease inhibitors used to treat HIV infection. The anti-neoplastic and immunomodulating agents with clinical trials are enzalutamide, methotrexate, imatinib, ruxolitinib, ibrutinib, and duvelisib. Enzalutamide had an AUROC affinity of 0.983 on CTSS, and it is an androgen receptor inhibitor to treat prostate cancer; methotrexate had an AUROC affinity of 1.0 on EGFR, and it is an antimetabolite used to treat breast cancer, lung cancer, head and neck cancer, and non-Hodgkin’s lymphoma; imatinib had an AUROC affinity of 1.0 on EGFR, and it is a BCR/ABL kinase inhibitor used to treat chronic myeloid leukemia, acute lymphoblastic leukemia, and gastrointestinal stromal tumors; ruxolitinib had an AUROC affinity of 1.0 on EGFR, and it is an inhibitor of JAK1/2 to reduce the hyperinflammation during cytokine storm in thrombocythemia myelofibrosis; ibrutinib had an AUROC affinity of 1.0 on EGFR, and it is an inhibitor of the Bruton tyrosine kinase causing protection against immune-induced lung injury; and duvelisib had an AUROC affinity of 1.0 on EGFR, and it is a PI3K inhibitor involved in the immune homeostasis restoration and viral replication inhibition. Finally, the anti-hemorrhagic agent with clinical trial was fostamatinib, which had an AUROC affinity of 1.0 on EGFR, and it is an inhibitor of spleen tyrosine kinase used to treat chronic immune thrombocytopenia (Supplementary Table S10; Wishart et al., 2018).

## Discussion

Since the finding of patient zero in China, a wide spectrum of clinical manifestations has been discovered, as we have understood the COVID-19 disease. The most common initial symptoms are cough, fever, anorexia, and dyspnea (Wang D et al., 2020; Berlin et al., 2020). The most common clinical features in severe COVID-19 patients are dyspnea, severe hypoxemia, lung edema, respiratory failure, ARDS (Montenegro et al., 2020), lymphopenia (Terpos et al., 2020), cardiac arrhythmias, rhabdomyolysis, hyperferritinemia, intravascular coagulopathy (Fogarty et al., 2020), and pulmonary thromboembolism (Rotzinger et al., 2020). Also, it has been observed that 15% of patients required supplemental oxygen (Young et al., 2020), and 5% of patients required mechanical ventilation. In addition, the smaller percentage of patients who required mechanical ventilation suffered comorbidities that lead to sepsis and septic shock (Rhee et al., 2020). Nowadays, it is known that SARS-CoV-2 is capable of reaching other organs depending on the host (Yang W et al., 2020). Different studies worldwide refer that clinical presentation vary between individuals, presenting manifestations not only respiratory tract infection, but also blood, skin, kidney, liver, ocular symptoms, neurologic signs, among others (Adhikari et al., 2020; Wang Q et al., 2020). Therefore, it is necessary to continuously review the reports on clinical manifestations in order to get to know the behavior of this disease as well as to think over the physiopathological mechanisms that allows us to better understand the related complications (Gupta et al., 2020; Wadman et al., 2020).

The effective immune response of the host, including the innate and adaptive ones, against SARS-CoV-2 seems to be essential to control and solve the infection. However, the clinical seriousness of COVID-19 could be associated to the excessive production of pro-inflammatory cytokines, known as ‘cytokine storm’ (Fajgenbaum and June, 2020; Hussman, 2020), or to the excessive production of bradykinin peptides, known as ‘bradykin storm’ (Garvin et al., 2020). This clinical paradigm is still to be figured out, and that is why the effective treatment is still uncertain. It is indispensable to understand the immunological responses that are triggered off since the beginning of the infection with SARS-CoV-2, so as to make progress in search of effective therapeutic strategies.

Innate immune response executes the first line of antiviral defense and is essential to obtain immunity against viruses (Zhong et al., 2020). Pattern recognition receptors (PRRs), codified by germline DNA, are responsible for recognizing widely common molecular patterns shared by pathogens of a certain group. Single-stranded and double-stranded viral RNAs produced during the replication phase of SARS-CoV-2 are recognized by endosomal TLRs (TLR7 and TLR8 or TLR3, respectively) and cytosolic RIG-I like receptors (RLRs), mainly RIG-I and MDA-5. After PRR engagement, downstream signaling pathways trigger the activation and nuclear translocation of key transcription factors, such as NF-kB, AP-1 and interferon regulatory factors (IRFs), and the ensuing expression of inflammasome activation and anti-viral cytokines (Lee et al., 2020). Among the most relevant cytokines we can find interleukins (IL-1, IL-6, and IL-18), pro-inflammatory TNF-α and TNF-ß, and type I and III IFNs (Blanco-Melo et al., 2020; Herold et al., 2020; McKechnie and Blish, 2020). Consequently, cytokines induce antiviral processes potentiating the innate and adaptive immune responses, limiting CoVs replication capacity and inducing the elimination of the virus cell reservoirs (Channappanavar et al., 2019; Blanco-Melo et al., 2020). However, CoVs have developed mechanisms of immune evasion where viral factors inhibit viral recognition by PRR sensing, and cytokine expression and secretion. Individuals with severe COVID-19 have demonstrated remarkably impaired type I IFN values as compared to mild patients (Hadjadj et al., 2020), and the interferon-induced overexpression of ACE2 may be involved (Ziegler et al., 2020).

Mucosal immune responses against viruses are orchestrated by myeloid cells such as macrophages, conventional DCs, plasmacytoid DCs, and monocyte-derived DCs (Guilliams et al., 2013). Accumulating evidence suggests that deregulation of myeloid cell-mediated responses potentially triggers lymphopenia, cytokine release syndrome, acute respiratory distress syndrome (Mehta et al., 2020), and pathogenic inflammation with high level secretion of IL-6, IL-2, IL-7, IFN-ɣ, IFN-I, and type III IFNs (Shi et al., 2019) in COVID-19 patients with severe clinical manifestations.

Innate lymphoid cells (ILCs) are lymphoid-like immune cells that lack the expression of rearranged antigen receptors. The non-cytotoxic group I, II, and III ILCs and the cytotoxic natural killer (NK) cells form the ILC family (Vivier et al., 2018). Several clinical data have reported that NK cells decrease in peripheral blood of severe patients (Song et al., 2020; Yu et al., 2020). An *in vitro* study has identified that the CXCL9-11 chemokines are overexpressed in lung cells infected with SARS-CoV-2, suggesting that the CXCR3 signaling pathway drives NK cells from peripheral blood to lungs in COVID-19 patients (Liao M et al., 2020). In addition, NK cells have the quality to induce lysis of infected cells causing severe hypoxemia and contributing to the cytokine storm resulting in ARDS.

T cells are involved in fundamental processes in viral infections. CD8 T cells eliminate infected cells and CD4 T cells help B cells for antibody production. Nevertheless, immunopathology is generated when T cells are dysregulated. Several reports have shown that moderate to severe COVID-19 patients with lymphopenia drastically reduce CD8 T cell and CD4 T cells in peripheral blood (Nie et al., 2020; Wen et al., 2020; Zeng et al., 2020). T cells reduction in the blood is also a contribution of mechanisms such as inflammatory cytokine milieu, which is why lymphopenia has a correlation with TNF-α, IL-6, and IL-10 (Diao et al., 2020; Wan et al., 2020). Conversely, clinical reports have shown that convalescent patients have low pro-inflammatory cytokine levels paired with restored bulk T cell frequencies (Diao et al., 2020).

The humoral immune response plays a main role in the clearance of cytopathic viruses and its memory response prevents reinfection. According to Huang *et al.* and Wu *et al.*, IgM, IgA, and neutralizing IgG antibodies can be detected in 12, 14 and 10–14 days, respectively, after symptom onset on average, suggesting that SARS-CoV-2 causes a robust B cell response in the majority of COVID-19 patients (Wu F et al., 2020; Huang et al., 2020). Indeed, antibodies binding the RBD of the S glycoprotein can have neutralizing properties, blocking virus interactions with the human protein receptor ACE2 (Ju et al., 2020), thereby inhibiting/preventing target cell infection. The B cell response to SARS-CoV-2 protects from the primary infection and extends immunity against reinfection due to memory B cells that can respond quickly by producing high affinity neutralizing antibodies. However, it is yet impossible to predict the duration of memory responses due to the timing of the COVID-19 pandemic.

There is currently a limited number of known risk factors that confer susceptibility to COVID-19. Several routine blood tests and immunological biomarkers have been suggested to classify patients with mild and severe symptoms. The routine blood test biomarkers currently suggested are lymphocyte count (Tan et al., 2020), neutrophil to lymphocyte ratio (Liu et al., 2020b), C-reactive protein (Ji et al., 2020), lactate dehydrogenase (Xiang et al., 2020), ferritin (Bataille et al., 2020), D-dimer and coagulation parameters (Zhou et al., 2020b), serum amyloid protein (Ji et al., 2020), N terminal pro B type natriuretic peptide (Gao L et al., 2020), platelet count (Qu et al., 2020), ultrasensitive troponin, and creatine kinase MB (Akhmerov and Marbán, 2020). On the other hand, immunological biomarkers associated with different COVID-19 outcomes are CD4^+^, CD8^+^, and NK cell count (Nie et al., 2020); PD-1 and Tim-3 expression on T cells (Diao et al., 2020); phenotypic changes in peripheral blood monocytes (Zhang D et al., 2020); expression levels of IP-10, MCP-3, IL-1ra (Yang Y et al., 2020); IL-6 (Chen et al., 2020), IL-8, IL-10, IL-2R, IL-1β (Gong et al., 2020), IL-4 (Fu et al., 2020), IL-18, granulocyte macrophage colony stimulating factor (GM-CSF) (Zhou et al., 2020a), IL-2, IFN-γ (Liu et al., 2020a), and anti-SARS-CoV-2 antibodies (Zhang B et al., 2020; Fu et al., 2020).

In this study, we performed proteomics, transcriptomics, and artificial neural network analyses to reveal potential therapeutic targets for drug repurposing to treat severe COVID-19. Firstly, we generated an immune system PPi network encompassing 1,584 nodes and 332,968 edges. Of them, 256 human proteins physically associated with SARS-CoV-2 proteins (Gordon et al., 2020) had high-confidence interactions with 1,390 immune system proteins. The degree centrality mean of the human proteins physically associated with SARS-CoV-2 was 23.6. GNB1, with the highest degree centrality, acts as a modulator in transmembrane signaling systems, including the GTPase activity (Gordon et al., 2020). The degree centrality mean of the immune system proteins was 44.5. UBA52, with the highest degree centrality, acts as a fusion protein that regulates ubiquitination of ribosome (Kobayashi et al., 2016). Lastly, the degree centrality mean of the phosphorylated proteins was 59.8. PIK3CA had the highest degree centrality and significant underexpression in SARS-CoV-2 infection in Vero E6 cells (Bouhaddou et al., 2020; Figure 2A; Supplementary Figure S1).

Overmyer *et al.* published a large-scale multi-omic analysis and identified 146 significantly expressed proteins in severe COVID-19 (Overmyer et al., 2020). We located these proteins and their high-confidence interactions in the immune system PPi network and subsequently generated the immune system PPi subnetwork encompassing 319 nodes and 5,308 edges. Of them, 26 significantly expressed proteins in severe COVID-19 (Overmyer et al., 2020) had high-confidence interactions with 49 human proteins physically associated with SARS-CoV-2 proteins, and with 281 immune system proteins. The degree centrality mean of the overexpressed proteins was 33.5. STOM, with the highest degree centrality, is located in cell membranes regulating ion channels and transporters. Loss of localization of the encoded protein is associated with hemolytic anemia shown in COVID-19 patients (Algassim et al., 2020). The degree centrality mean of the underexpressed proteins was 32.5. KNG1, with the highest degree centrality, is the precursor for bradykin synthesis, and is involved in the coagulation system dysfunction of severe COVID-19 (Sidarta-Oliveira et al., 2020). Lastly, the degree centrality mean of the phosphorylated proteins was 32.2, and PIK3CA had the highest degree centrality (Figure 2B).

SARS-CoV-2 employs a suite of virulent proteins that interact with key targets in host interactomes to extensively rewire the flow of information and cause COVID-19 (Vidal et al., 2011; Pan et al., 2016; Kumar et al., 2020). Although it has been shown that hubs of high-degree nodes are targets of numerous human viral (Calderwood et al., 2007; De Chassey et al., 2008; Gulbahce et al., 2012; Pan et al., 2016; Huttlin et al., 2017), COVID-19 is a novel disease and requires more in-depth studies. Therefore, we performed a functional enrichment analysis to validate the correlation between the subnetwork proteins and COVID-19 signatures published in studies worldwide (Figure 3). After a manual curation of gene ontology terms, the most significant biological processes were neutrophil degranulation (Shen et al., 2020), granulocyte activation (Yang L et al., 2020), myeloid leukocyte mediated immunity (Chen and John Wherry, 2020), inflammatory response (Jose and Manuel, 2020; Merad and Martin, 2020), blood coagulation (Vinayagam and Sattu, 2020), T-cell activation(Chen and John Wherry, 2020), response to interferon-gamma (Hu et al., 2020), platelet degranulation (Kuchi Bhotla et al., 2020), and acute inflammatory response (Manjili et al., 2020). The most significant KEGG pathways were chemokine signaling pathway (Chua et al., 2020), coagulation cascade (Overmyer et al., 2020), and antigen presentation (Li X et al., 2020). Lastly, the most significant Reactome signaling pathways were neutrophil degranulation (Wang J et al., 2020), innate immune system (Ahmed-Hassan et al., 2020), hemostasis (Liao D et al., 2020), signaling by VEGF (Kong et al., 2020), insulin-like growth factor (Winn, 2020), and platelet degranulation (Overmyer et al., 2020).

According to Buccitelli & Selbach (Buccitelli and Selbach, 2020), proteomics and transcriptomics typically show reasonable correlation, and integrating both types of data can reveal exciting biology and gene expression patterns. In light of this approach, the ‘COVID-19 Studies’ section of the Alexandria Project represents a large effort to characterize this immunopathology from a transcriptomics view. Ziegler *et al.* analyzed human scRNA-seq data to uncover potential targets of SARS-CoV-2 amongst tissue-resident cell subsets. They discovered *ACE2* and *TMPRSS2* co-expressing in goblet cells from nasal passage cells, type II pneumocytes from lung epithelial cells, and absorptive enterocytes from ileal epithelial cells (Ziegler et al., 2020). Therefore, after generating our immune system PPi network, we screened the 1,584 nodes into 10 nasal passage cells, 15 lung epithelial cells, and nine ileal epithelial cells to identify potential therapeutic targets for drug repurposing against COVID-19.

We found 75 significantly overexpressed molecules (Z-score > 2) in nasal goblet secretory cells (n = 5) (Figure 4), lung type II pneumocytes (n = 46) (Figure 5), and ileal absorptive enterocytes (n = 29) (Figure 6; Reimand et al., 2019). Subsequently, we analyzed the druggability of these 75 molecules (Methods section), and identified 25 potential therapeutic targets with ChEMBL ID and identified molecules with active/inactive interactions.

Meaningfully, these potential therapeutic targets not only were relevant in both the immune system PPi subnetwork and the scRNA-seq data, but also were involved in biological processes and signaling pathways related to severe COVID-19, such as neutrophil degranulation, blood coagulation or coagulation cascade, hemostasis, and platelet degranulation (Figure 7; Overmyer et al., 2020). Several studies worldwide have correlated these potential therapeutic targets with COVID-19. For instance, MAPK3 and EGFR showed kinase activity in the global phosphorylation landscape of SARS-CoV-2 infection according to Bouhaddou *et al* (Bouhaddou et al., 2020). CTSD, CD63, MKD, NFKBIA, MAPK3, STAT3, TNFSF10, F2RL1, HIF1A, NEU1, and EPAS1 were identified as significantly expressed targets in patients with severe COVID-19 according to Aschenbrenner *et al* (Aschenbrenner et al., 2020). C3, LDLR, CTSH, B4GALT1 and NFKBIA were significantly expressed targets in COVID-19 according to Alsamman & Zayed (Alsamman and Zayed, 2020). HSPA5 was associated with the viral entry, the endoplasmic reticulum stress, and anti-clotting agents according to Law *et al* (Law et al., 2020)*.* CD44 was involved in the extravasation cascade with significant expression in severe COVID-19 according to Chua *et al* (Chua et al., 2020). Basu *et al* found significant expression of the ITGA2 and ITGA3 integrins in COVID-19 patients (Basu et al., 2020). DDX3X was involved in the coronavirus-host protein-protein interactions according to Perrin-Cocon *et al* (Perrin-Cocon et al., 2020). Daniloski *et al* showed that ATP6AP1 induces shared transcriptional changes in cholesterol biosynthesis in human cells with SARS-CoV-2 infection (Daniloski et al., 2020). Lastly, CD74, CTSS and CTNNB1 were identified as potential targets for SARS-CoV-2 diagnosis and treatment according to Vastrad *et al* (Vastrad et al., 2020).

There is currently an urgent need for effective COVID-19 drugs. High-throughput screening for drug discovery has been important in finding antiviral drugs focused on the SARS-CoV-2 spike protein (Micholas and Jeremy, 2020) and the main protease (M^pro^), as detailed in our previous study (Tejera et al., 2020). However, computational structure-based drug discovery focused on immune system proteins is imperative to select potential drugs that, after being effectively analyzed in cell lines (i.e., African green monkey cells) and clinical trials, these can be considered for treatment of complex symptoms of COVID-19 patients. Drug repurposing offers a potentially rapid mechanism to deployment, since the safety profiles are known (Cabrera-Andrade et al., 2020; Phimister et al., 2020).

We performed fully connected deep neuronal networks to predict drugs with the highest affinities per target and multi-targets. We identified 47 approved drugs, 25 compounds under investigation, and 50 experimental compounds with the highest AUROCs for 15 (60%) of the 25 potential therapeutic targets. The best-predicted approved drugs were enrolled in ten different categories: anti-neoplastic and immunomodulating agents, anti-hemorrhagic agents, anti-inflammatory agents, anti-parathyroid agents, anti-viral agents, anti-oxidant agents, cardiovascular agents, central nervous system agents, growth hormone-releasing hormone, and antibiotics (see Results section and Figure 8).

There are around 4,000 clinical trials on COVID-19 using small molecules as single or combination agents with other anti-viral agents worldwide. Interestingly, 54 clinical trials currently correspond to 13 (27%) of the 48 best-predicted approved drugs found in our study (Figure 9). The cardiovascular agents implicated in the renin-angiotensin system are aliskiren, triamterene, and torasemide. Aliskiren and triamterene are renin inhibitors used to treat hypertension; and torasemide is used to treat edema associated with heart, renal, and hepatic failures. According to Garvin *et al.*, the renin-angiotensin system is an important pathway linked to hypertension and hypotension in COVID-19 patients because it maintains a balance of blood pressure (Garvin et al., 2020). The anti-viral agents are atazanavir, darunavir, and lopinavir. All of them are protease inhibitors used to treat HIV infection. According to Mahdi *et al.*, targeting of SARS-CoV-2 M^pro^ by HIV protease inhibitors might be of limited clinical potential due to the high concentration of drug required to achieve this inhibition. However, any potential beneficial effect in COVID-19 context might be attributed to acting on other molecular targets (Mahdi et al., 2020). The anti-neoplastic and immunomodulating agents are enzalutamide, methotrexate, imatinib, ruxolitinib, ibrutinib, and duvelisib. Enzalutamide is an androgen receptor inhibitor to treat prostate cancer; methotrexate is an antimetabolite that inhibits the dihydrofolate reductase and is used to treat breast cancer, lung cancer, head and neck cancer, and non-Hodgkin’s lymphoma; imatinib is a BCR/ABL kinase inhibitor used to treat chronic myeloid leukemia, acute lymphoblastic leukemia, and gastrointestinal stromal tumors; ruxolitinib is a Janus kinase 1 and 2 inhibitor that reduces the hyperinflammation during cytokine storm in thrombocythemia myelofibrosis; ibrutinib is an inhibitor of the Bruton tyrosine kinase causing protection against immune-induced lung injury; and duvelisib is a PI3K inhibitor involved in the immune homeostasis restoration and viral replication inhibition. According to Saini *et al.*, three hallmarks of cancer, namely immune dysfunction, inflammation, and coagulopathy are also seen in patients with SARS-CoV-2 infection, providing a biological rationale for testing anti-neoplastic agents for their ability to control the severe COVID-19 symptoms. However, these anti-neoplastic drugs should be evaluated carefully through well-designed and often novel trial platforms to avoid detrimental effects in future treatments (Saini et al., 2020). Finally, the anti-hemorrhagic agent, fostamatinib, is an inhibitor of spleen tyrosine kinase used to treat chronic immune thrombocytopenia. According to Kost-Alimova *et al.*, elevated mucin-1 (MUC1) protein levels predict acute lung injury and ARDS with poor clinical outcomes, and fostamatinib has been shown to reduce MUC1 abundance in a relevant pre-clinical model and has demonstrated safety profile in patients (Kost-Alimova et al., 2020; Tabassum et al., 2020).

Despite enormous scientific effort in drug repurposing studies to inhibit SARS-CoV-2 proteins or control severe COVID-19 symptoms, significant limitations exist. The main concern associated with drug repurposing studies involves the implementation of well-designed validation assays through clinical trials. Other main concerns are related to obtaining the correct therapeutic doses, safety results to avoid detrimental effects of repurposed drugs after treatments, and delivery capabilities worldwide (Parvathaneni and Gupta, 2020). All of this carried out counter clock due to the health emergency triggered by the pandemic. However, the positive side of this enormous scientific effort is to put forward recommendations for transforming today’s tools into solutions for future pandemics according to The National Symposium on Drug Repurposing for Future Pandemics, on behalf of the National Science Foundation.

The current COVID-19 pandemic offers a unique opportunity to strengthen mechanisms that promote the use of drug repurposing processes–considering the drug safety profile and the possibility of originate different adverse reactions in patients with distinct concomitant diseases–; inclusively, in the ongoing or future clinical trials, having the potential to reduce the time and costs for finding potential solutions to the current pandemic. Additionally, contributing to future analysis for high threat pathogens and rare diseases. This idea is welcomed by some other authors who conveyed on the potential of drug repurposing for common national and global health benefits (Yan, 2017). Between the several advantages of this process, the one which leads efforts to the use of the current information -on human pharmacology and toxicology-of safe and affordable generic drugs, is worth to remark. As also stated by Guy et al. (2020), along with this statement, there is the urge to motivate the transparency and compliance of the highest ethical principles for the conduction of studies, including as a key potential for drug repurposing, the visualization and sharing of negative results. Mainly, promoting and assuring that well-designed randomized clinical trials are timely implemented, especially during health emergencies and crises. In this sense, drug repurposing will be fulfilling its main objective: proposing potential, prompt, cost-effective, and safe solutions for the public and global health problems, with a human-centered approach.

The COVID-19 pandemic has evidenced that there is a strong urge to strengthen health systems with a major emphasis on health prevention and the major need, especially of low and middle income countries, to publicly invest on research and development. Consequently, the benefits of innovation and the results of research should be always available and affordable to anyone in need, to comply with the goal of public health (Røttingen et al., 2012). This is of particular importance during the current pandemic situation and on its aftermath.

From a global health perspective, initiatives directed to the improvement of rapid data sharing are critical during health emergency. This rapid sharing includes undoubtedly a transboundary collaboration founded on the principles of reliability and accuracy of the data (The Lancet, 2020). Meaningfully, for preventing potential new or existing pathogens to become high threats to human health and global security, non-commercial basic research on microorganisms should be assured. Additionally, introducing and promoting genomic epidemiology and strengthening global laboratory alliances would contribute to the national and global rapid detection and containment of outbreaks, as also promoted by the WHO. Accordingly, every country is sovereign and should guarantee the protection and regulation of the use of its biological resources, specifically working toward the Fair and Equitable Sharing of Benefits. Nevertheless, international conventions on the topic and national legislations should include fast track options for research on pathogens (Knauf et al., 2019). Relevantly, the links between human, environmental, and animal health - the One Health approach-are widely recognized to be effective toward the prevention and reduction of the emergence and re-emergence of potential pandemic agents (El Zowalaty and Järhult, 2020). This, not only pursuing to diminish the impact of epidemics or pandemics in the health systems, but also to underpin and reinforce economic, development, and social benefits.

## Data Availability

The datasets presented in this study can be found in the article and in online repositories. The names of the repository/repositories and accession number(s) can be found in the article/Supplementary Material.
